# Comparative Remarks

**Published:** 1885-06

**Authors:** 


### COMPARATIVE REMARKS.

*Colocynth and Staphisagria.*—*Colocynth* has great similarity to *Staphisagria*, not only in anger, with vexation and inclination to anger, but especially in abdominal colic, neuralgia, dysentery, and many other complaints. For this reason, they often act well after each other or in alternation.

*Plumbum*, similar to *Coloc.*, has an inclination to take the strangest attitudes and positions in bed.

*Calc. phosph.* and *Berberis* have both been given with great success to heal fistula in ano; both have, also, great similarity in their chest symptoms, particularly such as nearly always follow the surgical operation.

*Arsenic and Bryonia.*—*Ars.*, drinks little, but often; *Bry.*, drinks much, but not often. *Bry.*, eating often, but little at a time; *Ars.*, much eating at a time.

*Conium and Sulph. ac.*—If the desire to urinate is not soon enough satisfied: pain in the bladder, *Sulph. ac.*; pain in the kidneys, *Conium*. In ordinary cases, *Rhus tox.* is sufficient.

*Cuprum and Stramonium.*—*Nightly spasms.* Comp. *Staphisagria.* C. HG. in *J. of M. M.*

